# The National Insurance Bill

**Published:** 1911-10-21

**Authors:** 


### THE NATIONAL INSURANCE BILL.

### The Voluntary Hospitals Organised.

Mr. Lloyd George in his speech on the 14th instant made a great point as he was out to save the sufferings of his fellow-countrymen, and especially those who are poor and helpless, of having asked those who have knowledge and ideas to come and help him. He invited such organisations or groups to appoint " two, three, four of their men to meet him at the Treasury to consider the Bill " and explain how it could be made more perfect and so secure that it should fulfil to the utmost the requirements of all the interests and people affected. We welcome this reminder on the part of Mr. Lloyd George, and are glad to be able to inform him that the voluntary hospital managers are by inter-communication taking steps to secure united action in support of a few simple amendments to the National Insurance Bill as it stands, which will provide hospital benefit, give Mr. Lloyd George as Chancellor some additional economical safeguards, and lay the foundation for a perfected system of health administration by inter-communication with all existing institutions and means throughout the United Kingdom.

Letters will be found in another column in regard to certain matters intimately related to this bill. Mr. Godfrey Hamilton asks how the figures of £10,000,000 to £15,000,000 annually, as representing the amount the hospitals require in this country, are arrived at. If he refers to the speech he mentions he will find that the speaker was dealing with the question of the cost of substituting State hospitals for voluntary hospitals throughout the United Kingdom. The figures quoted will be found in the evidence given before the Poor Law Commissioners. What the cost of the scheme of State hospitals will actually be it is difficult to estimate, but one witness put it as high ultimately as £20,000,000 sterling a year, whilst the lowest estimate was £5,000,000 only, an impossible figure seeing that certainly more than £5,000,000 a year is provided already for hospital and institutional service for the sick under the voluntary system.

Mr. J. Courtney Buchanan raises the same point in a slightly different form. The figures contained in Burdett's " Hospitals and Charities " for 1911 relate only to some 1,260 institutions in 1908 which approximately administer to-day £5,000,000 sterling a year. As far as we can estimate, it is highly probable that the whole sum involved in the hospital and medical care provided through voluntary institutions in the British Islands is not far, if any, short of £7,000,000 a year. Anyone who examines the accounts of municipal or State authorities which have taken over great undertakings formerly under private administration, will be struck by the large additional expenditure in which this change often results, although in practice there may have frequently been no real improvement in the service, but rather the contrary, whilst the cost to the user has steadily gone up. Again, Mr. Lloyd George stated on the 14th instant that 15,000,000 persons will be insured under his Bill. That is to say, in round figures 36 per cent. of the entire population of the United Kingdom. As each of our correspondents is secretary to a London hospital we may add that that means that in the metropolis alone some two and a half million people or rather less will be insured. Let them compare this figure with the whole of the present hospital population of the Metropolis, and we think that they, in common with all experienced men of business, will agree, having the facts before them, that £15,000,000 is probably accurate so far as any forecast of the cost of State hospitals can, under present conditions, be made.

Dealing with Mr. Buchanan's next point, the effects of the Bill upon voluntary hospital income, we may state that the approximate figure of income in 1909 which is not secured will be found on page 177 of Burdett's " Hospitals and Charities," 1911. In the article published in The Hospital of October 7, entitled " Co-operation and Intercommunication," estimates are given which convince us, the more we examine them, that they may be taken as a forecast as accurate as possible of the actual amount of ordinary income which the voluntary hospitals must be prepared to lose unless hospital benefit is included in this Bill. Hospital benefit, if provided for in Clause 15, as we hope to show in another article, will strengthen immensely Mr. Lloyd George's Bill.

As to the number of persons who will be admitted to voluntary hospitals for treatment as " insured persons," should hospital benefit be not included in the Bill, our answer must be, no insured person can, without abuse, be admitted for free medical relief under the constitution of a voluntary hospital, because *every insured person will be clearly ineligible for free treatment.* The real danger of the moment arises from the complicated nature of the issues involved and the absence of accurate knowledge on the subject. The only way to save the voluntary hospitals from injury is for them to combine in every district, as we have recommended in previous articles. We are glad to have increasing evidence that steps are in process whereby the voluntary hospitals and similar agencies in every district will be rapidly organised. Everyone who believes in the voluntary hospitals—and who that has knowledge can fail to do so?—should each in his own district devote time and energy to the work of organising them locally. The sooner they wake up and complete this essential work the surer it will be that any risk of serious injury for the voluntary hospitals will be rendered practically impossible, by a second conference with the Chancellor of the Exchequer, when all the facts, carefully marshalled, can be clearly presented by the most knowledgeable speakers available.

